# Identifying Novel Drug Targets by iDTPnd: A Case Study of Kinase Inhibitors

**DOI:** 10.1016/j.gpb.2020.05.006

**Published:** 2021-03-29

**Authors:** Hammad Naveed, Corinna Reglin, Thomas Schubert, Xin Gao, Stefan T. Arold, Michael L. Maitland

**Affiliations:** 1 Toyota Technological Institute at Chicago, Chicago, IL 60637, USA; 2 Department of Computer Science, National University of Computer and Emerging Sciences, Islamabad 44000, Pakistan; 3 2bind GmbH, D-93053 Regensburg, Germany; 4 King Abdullah University of Science and Technology (KAUST), Computational Bioscience Research Center (CBRC), Computer, Electrical and Mathematical Sciences and Engineering (CEMSE) Division, Thuwal 23955, Saudi Arabia; 5 King Abdullah University of Science and Technology (KAUST), Computational Bioscience Research Center (CBRC), Biological and Environmental Sciences and Engineering (BESE) Division, Thuwal 23955, Saudi Arabia; 6 Inova Center for Personalized Health and Schar Cancer Institute, Falls Church, VA 22042, USA; 7 University of Virginia Cancer Center, Annandale, VA 22003, USA

**Keywords:** Protein–drug interaction, iDTPnd, Kinase inhibitor, Drug-binding site signature, Drug repurposing

## Abstract

Current FDA-approved **kinase inhibitors** cause diverse adverse effects, some of which are due to the mechanism-independent effects of these drugs. Identifying these mechanism-independent interactions could improve drug safety and support **drug repurposing**. Here, we develop **iDTPnd** (integrated Drug Target Predictor with negative dataset), a computational approach for large-scale discovery of novel targets for known drugs. For a given drug, we construct a positive structural signature as well as a negative structural signature that captures the weakly conserved structural features of drug-binding sites. To facilitate assessment of unintended targets, iDTPnd also provides a docking-based interaction score and its statistical significance. We confirm the interactions of sorafenib, imatinib, dasatinib, sunitinib, and pazopanib with their known targets at a sensitivity of 52% and a specificity of 55%. We also validate 10 predicted novel targets by using *in vitro* experiments. Our results suggest that proteins other than kinases, such as nuclear receptors, cytochrome P450, and MHC class I molecules, can also be physiologically relevant targets of kinase inhibitors. Our method is general and broadly applicable for the identification of protein–small molecule interactions, when sufficient drug–target 3D data are available. The code for constructing the structural signatures is available at https://sfb.kaust.edu.sa/Documents/iDTP.zip.

## Introduction

Proteins that contain kinase domains are involved in numerous cellular processes including signaling, proliferation, apoptosis, and survival [1], [2]. The human kinome consists of more than 500 members [3]. These kinases have diverse sequences but a high degree of 3D structure similarity, particularly in the ATP binding pocket [4]. Kinases are the primary drug targets for the treatment of many cancers [5], [6], [7]. There are more than 30 FDA-approved small molecule kinase inhibitors that bind to kinase domains reversibly or irreversibly. The kinase inhibitors that bind reversibly can be categorized into four major types, based on the binding pocket conformation and the aspartate-phenylalanine-glycine (DFG) motif of the kinase activation loop controlling access to the binding pocket [8], [9]. Most of these inhibitors fall into the type I or type II categories. Type I kinase inhibitors bind to the active forms of kinase domains in an ATP-competitive manner, with the aspartate amino acid facing into the active site. Type II kinase inhibitors, on the other hand, bind to the inactive forms of kinase domains with the aspartate residue facing outside the active site [8], [9]. Given that the ATP-binding site has necessarily conserved features across most kinase domains, several kinase inhibitors interact with the human kinome broadly and are not very selective; on average, 26% (135) of all human kinases interact with one or more kinase inhibitors included in this study [10], [11]. This broad reactivity affects the inhibitor’s efficacy and toxicity [12], [13], [14]. Therefore, predicting kinase inhibitor targets or off-targets is central for the rapid and cost-efficient development of inhibitors, as it allows a better understanding of a drug’s adverse effects and exploration of the drug repositioning opportunities [15].

Recent studies have estimated that many unintended targets of approved drugs are yet to be discovered [16], [17], and this mechanism-independent binding leads to toxicity [14]. It is difficult to determine whether the unexpected adverse effects of new drugs especially kinase inhibitors are due to their binding to unexpected targets or unknown relationships between their intended targets and the function of a complete human organ system. Therefore, predicting mechanism-independent binding sites could enhance early evaluation of a compound’s specificity and hence the likelihood for specific clinical consequences.

Computational methods have increasingly been used for hit identification and lead optimization [18]. These methods fall into four categories: methods that use 1) binding site structure, 2) gene expression, 3) ligand structure, and 4) a combination of all above. Structure-based methods employ binding site similarity and/or molecular docking [19], [20], [21], [22]; expression-based methods use the expression level changes of proteins that results from the drug activity [23], [24], [25], [26], [27]; ligand-based methods utilize the structural and chemical properties of a drug [28], [29], [30]; and hybrid methods combine two or more types of data [31], [32], [33], [34], [35]. In addition, novel targets for drugs have also been identified by comparing adverse effects [36] and by using genome-wide association studies [37].

In this study, we propose a computational method for large-scale discovery of new drug targets, named iDTPnd, which markedly improves our previous methodology [38] by incorporating a negative structural signature (*i.e.*, a conserved structural signature in the kinases that is known not to interact with the respective drug). We now also provide a docking-based interaction score along with its statistical significance. In a blind test of five FDA-approved kinase inhibitors, we predict the known targets with 52% sensitivity and 55% specificity. This is a significant improvement compared to a baseline model based on sequence similarity and to a recently published study [17], which reports a precision of 30% and a recall of 27% with an estimated false positive rate of 70%. In addition, our methodology is generic and can be used broadly for all types of small molecule drugs for which sufficient 3D structures of known targets (∼30) are available. We also validate 10 predicted interactions through *in vitro* experiments. It is important to note that our predictions are not limited to kinases.

## Method

### Dataset

We extracted the positive and negative datasets from the kinome scan assay of Davis et al.’s work [11]. Five kinase inhibitors were selected for this study, each of which had one co-crystallized structure with its target and had at least 30 known targets with experimentally determined structures (apo or bound to other entities) available (Table S1). Redundancy reduction was carried out as follows: for the positive dataset, a cut-off of 70% sequence identity was used for all structures that were not bound to the respective drug, and all co-crystallized structures were included (usually 1–4) in the positive dataset; for the negative dataset, a stricter cut-off of 60% sequence identity was used, as we had a relatively larger dataset. We did not do redundancy reduction between the positive and negative datasets. As structural databases are growing exponentially, the number of drugs to which the method can be applied is expected to increase significantly. The total structures deposited in Protein Data Bank (PDB) at the end of 2011 were 77,452, while increased to 150,593 on April 4, 2019 (https://www.rcsb.org/). This means that the number of structures has almost doubled in 7 years. Similarly, in case of the membrane proteins which are the targets of more than 60% marketed drugs, we had 328 structures in 2011, while this number increased to 876 on April 4, 2019 (https://blanco.biomol.uci.edu/mpstruc/). Therefore, we expect our method to be applicable for broader set of studies going forward.

### Sequence similarity baseline model

The sequence similarity baseline model used nearest neighbor algorithm to allocate a protein to the interacting or non-interacting cluster. Using leave-one-out cross validation, global pairwise sequence similarity (not identity) was calculated between the left-out protein and all other proteins. The left-out protein was assigned to the cluster that contained the protein with the maximum pairwise global sequence similarity to the left-out protein. If none of the protein pairs had a global sequence similarity >0.6, a label was not assigned to the left-out protein.

### Structural signature construction

The flowchart of our method is shown in Figure S1. Briefly, CASTp webserver was used to extract the pocket that the drug binds to [39], referred to as the ‘binding pocket’ from here on. Sequence order-independent alignment was used to find the pocket similar to the binding pocket [40], [41] using the distance function described below. We extracted the conserved (positive and negative) structural signatures by applying pairwise sequence order-independent structure alignment followed by hierarchical clustering.

Score=StructuralScore+α×SequenceScore

$$\mathrm{Structural}\;Score=RMSD\times N^{\left(-1/3\right)}$$

$$\mathrm{Sequence}\;Score=1-\left(\mathrm{Sequence}\;Similarity/Best\;Sequence\;Similarity\right)$$

$$\mathrm{Sequence}\;Similarity=\sum_{i}\left(AtomFreq_{i}+ResFreq_{i}\right)$$

$$\mathrm{Best}\;Sequence\;Similarity=\sum_{i}\left(MaxAtomFreq_{i}+MaxResFreq_{i}\right)$$

Our method is not sensitive to the exact value of *α* as long as it is close to 1. The *α* can be adjusted according to the empirical insight from the data. In this study, we used *α* = 1.2. *RMSD* is the root mean square distance, *N* is the number of positions aligned, ${\mathrm{AtomFreq}}_{i}$/${\mathrm{ResFreq}}_{i}$ represents the frequency of atom/residue aligned at position *i*, ${\mathrm{MaxAtomFreq}}_{i}$/${\mathrm{MaxResFreq}}_{i}$ represents the maximum frequency of any atom/residue aligned at position *i*, and the summation is over all aligned positions. Every position in the signature is present in at least 50% of the structures. To achieve a minimalistic structural signature, preservation ratio cut-off is increased if the number of atoms in the signature is more than 100. While combining the positive and the negative structural signatures, predicted targets are those that have a better positive score than the negative one (Score_positive_ – Score_negative_ < 0).

### MicroScale thermophoresis

The predicted protein targets, human leukocyte antigen A (HLA-A; Catalog No. TP300661, Acris Antibodies GmbH, Herford, Germany), human leukocyte antigen B (HLA-B; Catalog No. TP310631, Acris Antibodies GmbH), mitogen-activated protein kinase-activated protein kinase 2 (MAPKAPK2; Catalog No. 14–337, Merck Millipore, Darmstadt, Germany), cAMP-specific 3ʹ,5ʹ-cyclic phosphodiesterase 4B (PDE4B; Catalog No. 11527-H20B-20, Hölzel Diagnostika Handels GmbH, Cologne, Germany), protein kinase C eta type (PKCη; Catalog No. ab60849, Abcam, Regensburg, Germany), estrogen receptor α (ERα; Catalog No. USC-RPB050HU01-50, Biozol, Munich, Germany), cyclin-dependent kinase 2 (CDK2; Catalog No. ABIN2003156, Antikoerper, Aachen, Germany), and tyrosine-protein kinase ITK/TSK (Catalog No. BPS-40445, Biomol, Hamburg, Germany) were labeled by using NHS chemistry with the help of an NT647-labeling kit (NanoTemper Technologies, Munich, Germany). In an initial step, the Tris-containing storage buffers were exchanged by the MicroScale thermophoresis (MST) labeling buffer as indicated by the manufacturers in order to avoid labeling primary amines in Tris. After addition of a two-molar excess of reactive NT647 dye to the respective target protein, the reaction was incubated in the dark for 30 min. After this, the unbound dye was removed using a size exclusion column as indicated by the manufacturers. Using the buffer containing 1× PBS pH 7.5, 0.1% pluronic F127, and 2% DMSO did not result in aggregation or sticking effects for HLA-A, HLA-B, MAPKAPK2, CDK2, PKCη, ITK, and ERα. Standard, premium, and hydrophobic capillary types were tested for non-specific sticking of the proteins to the glass surface. HLA-A, HLA-B, and ERα showed sticking in standard capillaries but no sticking in premium capillaries. Hence, premium capillaries were used for the further experiments for these proteins. The other proteins remained in solution in standard capillaries. PDE4B showed aggregation in all conditions and hence was not tested further. The LED power was set to 10%–25% to obtain optimal signal intensities. The laser power was identified being optimal at 40% or 80%. Changes in amplitudes between the lower and upper binding curve plateau of more than 4 units and a signal-to-noise ratio of more than 6 were considered significant for binding events. Each experiment had two replicates.

## Results and discussion

### Structural signatures

**Figure 1:**
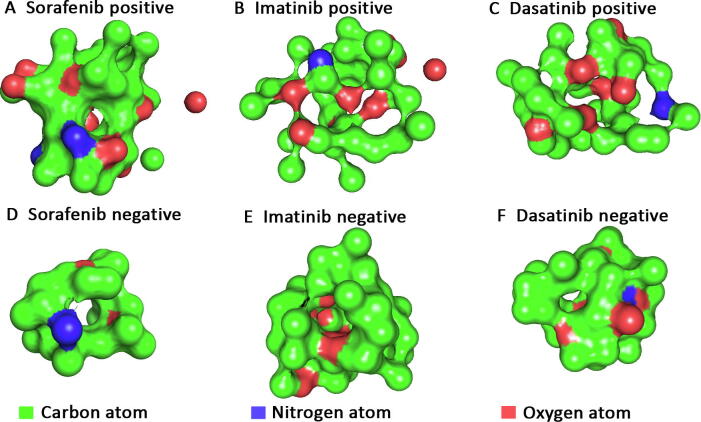
**Structural signatures** 3D structural signatures (positive and negative) of sorafenib (**A** and **D**), imatinib (**B** and **E**), and dasatinib (**C** and **F**). The color of each position represents the atom with the highest frequency. Carbon, oxygen, and nitrogen atoms are labeled in green, red, and blue, respectively.

We first constructed the structural signature from the positive dataset [11], using sequence-independent structure alignment, hierarchical clustering, and a probabilistic scoring function (Figure 1A–C; see Method for details). This method (named iDTP) has been successful in representing the binding pocket signature of 11 metabolites (drugs) in our earlier study [38]. However, in this study, we found that the positive structural signature alone is not sufficient to distinguish targets from non-targets in the case of kinase inhibitors. Indeed, for the five drugs (sorafenib, imatinib, dasatinib, sunitinib, and pazopanib), we obtained an average sensitivity and specificity of 31% (±34%) and 78% (±25%), respectively, using the cut-off of 0.85 specified in our previous study [38] (Table 1, Table S2). This large standard deviation (SD) suggests that the iDTP’s performance based on a positive signature is not very reliable when the targets and non-targets share significant similarity. This might be due to a combination of reasons, 1) the ATP binding pocket is structurally conserved across the kinase domains, 2) the orientation of the DFG motifs differs across the kinase domains, and 3) there are subtle changes in the binding interaction of the kinase inhibitors with kinase domains [42]. To resolve these issues, we built a structural signature from the negative dataset [11] dubbed “negative signature”, using the same procedure used for the positive dataset (Figure 1D–F). The pocket that was most similar to the binding pocket from each structure was used to construct the negative signature. This is similar to the well-established practice of using near-native decoys to improve the docking-based scoring functions [43]. The numbers of structures used to make the positive signatures, negative signatures, and the preservation ratio for each signature are given in Table S3. A protein was considered as a target only when one of its top 3 largest pockets had a better (lower) score (as defined in Method), after aligning with the positive structural signature as compared to the score of the same pocket aligning with the negative structural signature (Score_positive_ – Score_negative_ < 0). Combining the positive and negative structural signatures helped improve the performance of the methodology significantly across all the kinase inhibitors (Table 1). Using a dataset of all kinases with experimentally determined structures and five FDA-approved kinase inhibitors, we predicted their known targets with an average sensitivity of 52% (ranging from 42% to 68%) and an average specificity of 55% (ranging from 50% to 59%) in 5-fold cross-validation tests. To evaluate the effect of sequence similarity in the dataset, we constructed a baseline model using sequence similarity (see Method). Using this baseline model, we only assigned 47% of the proteins to either the interacting or non-interacting cluster with an average sensitivity of 32% (±5%) and an average specificity of 26% (±9%).

**Table 1:** P**erformances of three methods on five kinase inhibitors**

**Drug**	**iDTP (positive signature)**		**iDTPnd (positive + negative signature)**		**Baseline model (sequence similarity)**	
	**Sensitivity**	**Specificity**	**Sensitivity**	**Specificity**	**Sensitivity**	**Specificity**
Sorafenib	81%	33%	68%	59%	42%	31%
Imatinib	3%	94%	55%	54%	32%	17%
Dasatinib	2%	99%	42%	57%	29%	18%
Sunitinib	63%	67%	46%	50%	26%	23%
Pazopanib	4%	95%	50%	55%	32%	40%
Average (±SD)	31% (±34%)	78% (±25%)	52% (±9%)	55% (±3%)	32% (±5%)	26% (±9%)

*Note*: SD, standard deviation.

A previous study has shown that a synthetic negative dataset can be constructed to assess the specificity of methodologies [38]. Here, we found that using a synthetic negative dataset could severely over-estimate specificity. For example, using the positive signature alone for sorafenib, the specificity on the negative dataset constructed by iDTP [38] was 65%, while the specificity was 33% on the negative dataset based on previous experimental results [11]. In the case of kinase inhibitors, experimental kinome profiling companies like DiscoverX have explored kinase inhibitor interactions extensively [11]. According to our dataset, the five kinase inhibitors chosen in this study interacted with 26% of the kinases (on average) and the rest were considered as true negatives. Any novel interactions discovered from these negative examples are therefore non-trivial as the model was trained to treat them as negative. The probability of discovering three novel interactions amongst the top 10 predictions can be determined by using combinatorics and is very small (less than 1%).

### Identification of new targets

In order to identify new drug targets, we extracted the top 3 pockets (largest volume) of structures deposited in PDB using CASTp [39]. We then aligned the structural signatures (both positive and negative) of each drug with these pockets. Similar to self-validation tests, a protein was considered as a potential target only when one of the pockets had “Score_positive_ – Score_negative_ < 0”. Flexible docking option of SwissDock was further used to predict the interacting strength between the drug and the potential target [44]. To address the relatively high false positive rate and the imperfections in the scoring functions associated with docking methods, we measured the significance for these binding scores. The significance was calculated by comparing the binding scores obtained for potential targets of a drug with the binding scores of 100 random protein structures with the respective drug. The size of random samples can be increased to improve statistical significance at the cost of significant computational time. The random structures were sampled from a list of protein structures that have less than 60% identity with the positive and negative datasets. The significance measurement enabled us to identify more promiscuous compounds such as gefitinib, where even the targets with most favorable docking score have an unfavorable significance measure. This supported that the compound is unusually sticky and is interacting with many proteins with high probability. Therefore, we excluded gefitinib from our study. Table 2 gives the top 10 predicted targets of sorafenib after redundancy reduction using the PISCES webserver [45]. The top 10 predicted targets for the other four kinase inhibitors are given in Tables S4–S7. Our ranking consists of two steps. The first step identifies all protein targets for which “Score_positive_ – Score_negative_ < 0”. The second step sorts the targets in an ascending order with respect to the docking score.

**Table 2:** **Top 10 predicted targets of sorafenib**

**Target**	**Score_positive_**	**Score_negative_**	**Score_positive_ − Score_negative_**	**Docking score**	**Significance measure**
PDE4B	0.69	0.83	−0.14	−167.63	<1%
SRC	0.70	1.17	−0.47	−92.22	2%
HLA-B	0.67	0.86	−0.19	−85.80	2%
HLA-A	0.69	0.75	−0.06	−73.87	3%
MAPK1	0.65	0.99	−0.34	−72.66	4%
MAPKAPK2	0.64	0.86	−0.22	−50.03	9%
QPRT	0.65	0.76	−0.11	−40.77	9%
FLG	0.7	0.72	−0.02	−23.11	11%
FECH	0.68	0.9	−0.22	−13.99	14%
PKCη	0.58	0.78	−0.20	−11.71	14%

*Note*: The predicted targets have less than 60% sequence similarity with the known targets of sorafenib. Proteins that do not contain a kinase domain are shown in bold. PDE4B, cAMP-specific 3ʹ,5ʹ-cyclic phosphodiesterase 4B; SRC, proto-oncogene tyrosine-protein kinase Src; HLA-B, human leukocyte antigen B (57:01.I80T); HLA-A, human leukocyte antigen A (02:03); MAPK1, mitogen-activated protein kinase 1; MAPKAPK2, mitogen-activated protein kinase-activated protein kinase 2; QPRT, nicotinate-nucleotide pyrophosphorylase; FLG, filaggrin; FECH, ferrochelatase, mitochondrial; PKCη, protein kinase C eta type.

### Experimental validation

**Figure 2:**
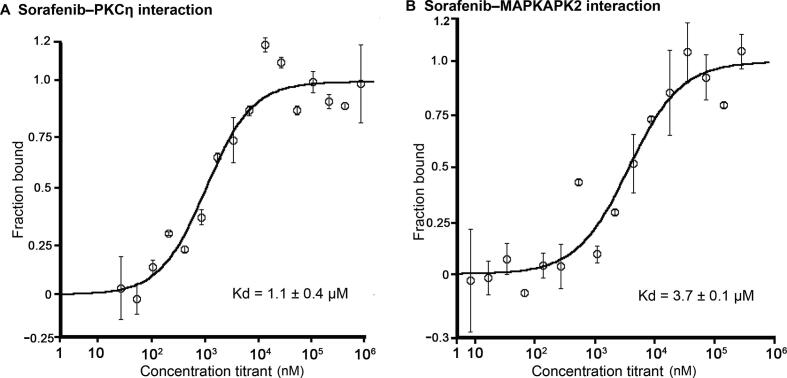
**Interactions of sorafenib with PKCη and MAPKAPK2** **A.** Binding curve of sorafenib with PKCη. **B.** Binding curve of sorafenib with MAPKAPK2. The predicted interactions of sorafenib with PKCη and MAPKAPK2 proteins were experimentally verified through MST experiments. MST-derived binding curves of NT647-labeled PKCη and MAPKAPK2 to sorafenib were plotted as a function of sorafenib concentration. Data are represented as mean ± SD (*n* = 2). MST, MicroScale thermophoresis.

To provide an experimental validation of iDTPnd, we first chose five predicted targets (PDE4B, HLA-A, HLA-B, MAPKAPK2, and PKCη) of sorafenib to test, considering the target’s ranking in our results as well as the availability and cost of the purified protein. We used MST experiments to test the predicted interactions *in vitro* (see Method for details). The results showed that PKCη and MAPKAPK2 interacted with sorafenib with dissociation constant (Kd) values of 1.1 ± 0.4 μM and 3.7 ± 0.1 μM, respectively (Figure 2). Considering that affinities of sorafenib to its primary targets are comparable, most of which are within the range from 100 nM to 1 μM [11], the interactions of sorafenib with PKCη and MAPKAPK2 might be pharmacologically relevant and hence valuable for medicinal chemists. Similarly, our results also showed that sorafenib interacted with HLA-A and HLA-B with Kd values between 300 μM and 600 μM. It was speculated but plausible that, due to the intracellular accumulation of sorafenib in some cell types and the wide diversity of HLA isotypes, this weak *in vitro* interaction could be clinically relevant [46], [47]. For example, severe drug-specific adverse effects of sorafenib have been reported to be associated with HLA-A24 sub-type of HLA-A [48]. Moreover, the immune system is compromised while taking sorafenib, and flu vaccination is not recommended during this period [49]. Finally, HLA-B is known to directly interact with a small molecule drug [50]. PDE4B was the top target predicted in our study, but it showed aggregation under all test conditions and the results remained inconclusive. Next, as ITK was the top ranked prediction of imatinib, we tested their interaction to see if iDTPnd works for other kinase inhibitors besides sorafenib. In MST assays, imatinib interacted with ITK with a Kd of 550 ± 120 nM. The confirmation of this interaction suggested that our method detects previously unrecognized direct physical interactions, and so we proceeded to evaluate predicted interactions for additional kinase inhibitors.

### ERα

ERα is a nuclear receptor that is activated by estrogen and is important for hormone/DNA binding and transcription activation [51]. The role of ERα in breast cancer is well documented with nearly 70% of newly diagnosed breast cancers being ER positive (cancer cells grow in response to the hormone estrogen) [52]. ERα is one of the primary targets of tamoxifen, an FDA-approved drug for breast cancer treatment [53]. In our study, ERα was ranked 3rd, 9th, and 10th among the predicted targets for sunitinib, pazopanib, and dasatinib, respectively (Table 3). We used MST to test our predictions *in vitro*. We also included sorafenib and imatinib in our experiments to test our false negative rate.

**Table 3:** **Interactions between kinase inhibitors and ERα**

**Drug**	**Score_positive_**	**Score_negative_**	**Score_positive_ − Score_negative_**	**Docking score**	**Random chance**	**Binding affinity** **(mean ± SD)**
Sunitinib	0.52	0.74	−0.22	−154.7	7%	14.7 ± 5.7 nM
Dasatinib	0.51	0.75	−0.24	−52.83	8%	1.2 ± 0.5 μM
Pazopanib	0.57	0.76	−0.19	−43.23	3%	3.2 ± 1.0 μM

**Figure 3:**
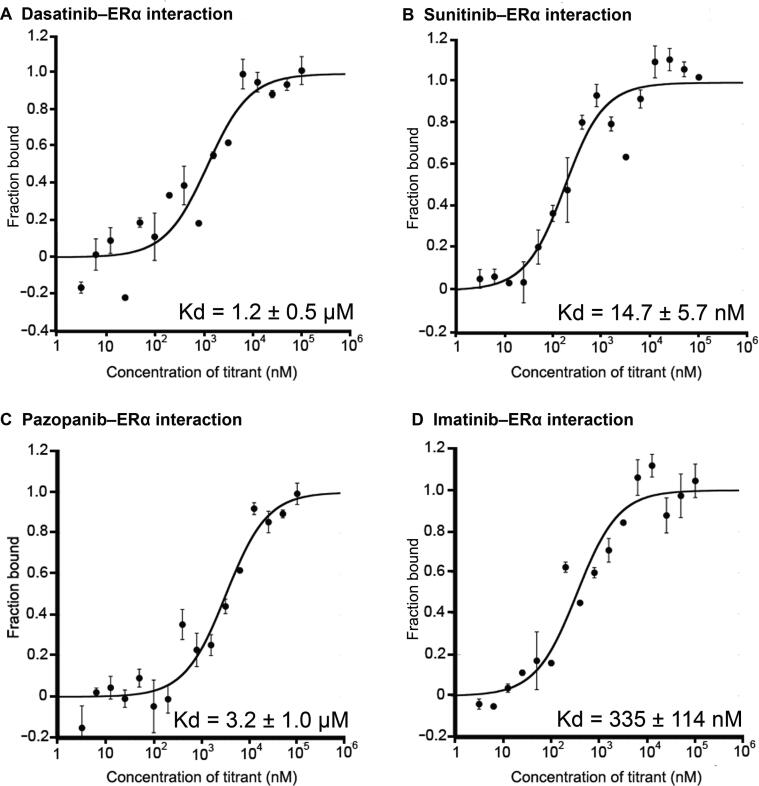
**Interactions of kinase inhibitors with ERα** **A.** Binding curve of dasatinib with ERα. **B.** Binding curve of sunitinib with ERα. **C.** Binding curve of pazopanib with ERα. **D.** Binding curve of imatinib with ERα. MST-derived binding curves of NT647-labeled ERα to ligands were plotted as a function of ligand concentration. Data are represented as mean ± SD (*n* = 2). ERα, estrogen receptor α.

Sunitinib, dasatinib, and pazopanib were found to interact with ERα with Kd values of 14.7 ± 5.7 nM, 1.2 ± 0.5 μM, and 3.2 ± 1.0 μM, respectively (Figure 3). While sorafenib did not interact with ERα as predicted at detectable levels in our setup, we found that imatinib bound to ERα with a Kd value of 335 ± 114 nM even though ERα was not predicted as a target for imatinib in our results, suggesting that iDTPnd does have some false negatives. In support, a recent case study has reported the response of a patient’s ER^+^/HER2^−^ breast cancer tumors to pazopanib after the tumors had developed resistance to endocrine therapy [54]. Although the study did not explore the mechanism of how pazopanib interacted with fibroblast growth factor receptors to amplify FGFR1 in the tumor, the direct interaction between pazopanib and ERα might have contributed to this clinical response. Another study has shown that dasatinib can block ERα-facilitated extranuclear actions that lead to metastasis [55]. This regulation can be due to the direct interaction between dasatinib and ERα. Sunitinib has also been reported to inhibit tumor growth in breast cancer cells [56]. Further studies are required to comprehensively understand the pharmaceutical effects of these interactions.

### CDk2

Cyclin-dependent kinases (CDKs) perform important roles in cell division cycle, transcription, differentiation, neuronal functions, and apoptosis [57]. Specifically, CDK2 has been implicated in prostate cancer, non-small cell lung cancer, and breast cancer [58], [59], [60]. Several CDK2 inhibitors have been developed to check aberrant CDK2 activity. Sorafenib has been shown to interact with CDK2 [11]. CDK2 also appeared as one of the top targets of dasatinib and imatinib in our *in silico* prediction. In our next round of MST experiments, we also included sorafenib as the positive control and used two other kinase inhibitors (sunitinib and pazopanib) to test our false negative rate.

**Figure 4:**
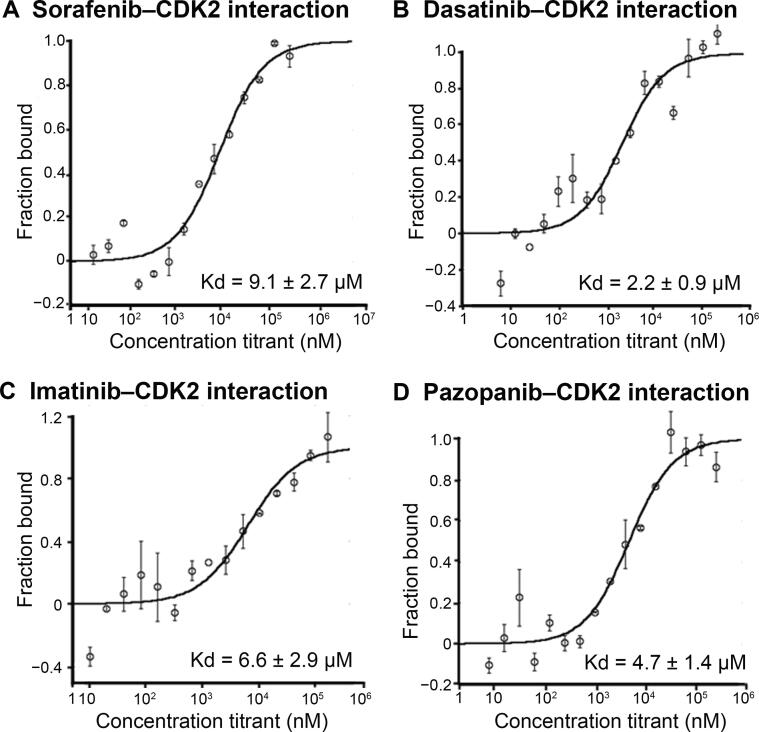
**Interactions of kinase inhibitors with CDK2** **A.** Binding curve of sorafenib with CDK2. **B.** Binding curve of dasatinib with CDK2. **C.** Binding curve of imatinib with CDK2. **D.** Binding curve of pazopanib with CDK2. MST-derived binding curves of NT647-labeled CDK2 to ligands were plotted as a function of ligand concentration. Data are represented as mean ± SD (*n* = 2). CDK2, cyclin-dependent kinase 2.

Dasatinib, imatinib, and sorafenib were found to interact with CDK2 with Kd values of 2.2 ± 0.9 μM, 6.6 ± 2.9 μM, 9.1 ± 2.7 μM, respectively (Figure 4). We found that pazopanib also interacted with CDK2 with a Kd value of 4.7 ± 1.4 μM even though CDK2 was not predicted as a target for pazopanib in our results (Figure 4). While sunitinib did not interact with CDK2 as predicted at detectable levels by iDTPnd, the interaction between CDK2 and dasatinib was indirectly supported by a previous study that showed selective modulation of CDK2 by dasatinib [61]. Our results indicate that this modulation is a direct result of the interaction between CDK2 and dasatinib.

### MHC class I proteins

**Figure 5:**
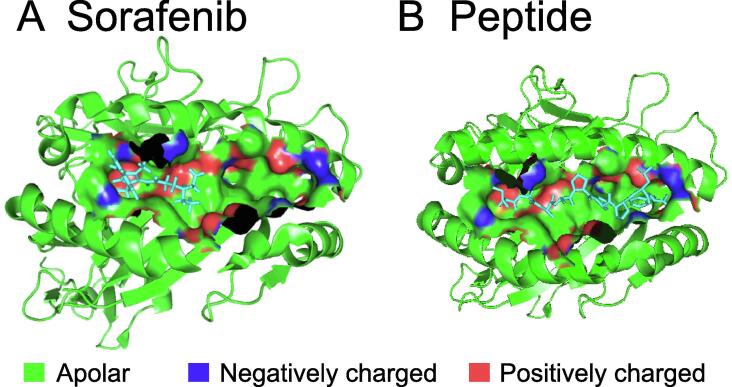
**Ligand-binding pocket of MHC class I proteins** **A.** Sorafenib bound in the pocket (determined by flexible docking). **B.** Peptide bound in the pocket (in the crystal structure). The extracellular ligand-binding groove of MHC class I proteins, taken from PDB 3BXN, is shown as green ribbon. In the ligand-binding pocket identified by iDTPnd, apolar atoms, negatively charged atoms, and positively charged atoms are shown in green, blue, and red, respectively. Peptide shown in (B) is a Cathepsin A signal sequence octapeptide.

We observed that MHC class I (HLA-A/HLA-B) proteins were predicted as potential targets for all kinase inhibitors used in this study except pazopanib. The cell surface of all nucleated cells contains MHC class I proteins in jawed vertebrates [62]. They bind peptides (formed due to degradation of cytosolic proteins) and display them to the cytotoxic T cells. Cytotoxic T cells bind the presented peptide and initiate an immune response on the recognition of an infected state. Peptide binding to the MHC class I proteins is the most selective step in the antigen presentation pathway. As of August 2016, there were 33 (sequence similarity <99%) experimentally resolved structures available for different alleles of MHC class I proteins. To further explore the interactions of kinase inhibitors with MHC class I proteins, we performed the flexible docking of all five kinase inhibitors with each of the 33 structures. The dominant interactions (determined using flexible docking) between the kinase inhibitors and the MHC class I proteins existed in the peptide-binding region (Figure 5), which was one of the two pockets identified by using the structural signatures. The interaction in the peptide-binding region was significant as it might change the peptides being presented to cytotoxic T cells as in the case of abacavir, which is FDA-approved for HIV treatment [63]. The second pocket identified was located between the two chains of the MHC class I proteins. Our results showed that with the exception of pazopanib, other four kinase inhibitors tested in this study directly interacted with many HLA alleles (13–31 out of 33) (Table 4). This interaction might compete with the peptides being presented to cytotoxic T cells. The direct interactions between the kinase inhibitors and MHC class I proteins might initiate an immune response responsible for the observed side effects.

**Table 4:** **Interactions between kinase inhibitors and MHC class I proteins (HLA alleles)**

**Drug**	**Best docking score**	**No. of structures showing favorable binding (out of 33)**
Sorafenib	−149.75	13
Sunitinib	−199.22	31
Imatinib	−126.73	15
Dasatinib	−188.48	18
Pazopanib	−157.97	2

### Cytochrome p450 enzymes

Cytochrome p450 enzymes (CYPs) are the most important enzymes involved in drug metabolism. They account for about 75% of the total metabolism. Most drugs are deactivated by CYPs, either directly or indirectly [64]. Drug metabolism by CYPs is a key reason of adverse drug interactions, as altered CYP enzyme activity can affect the metabolism and removal of drugs from the body [64]. In our study, CYP2E1 and CYP2A6 were among the top predicted targets of dasatinib, and CYP1A2 was among the top predicted targets of pazopanib (Tables S4 and S7). As CYPs play an important role in the drug metabolism, FDA tests these interactions before approving a drug. We found that the predicted interactions were indeed reported in the FDA Orange Books (Table 5) [65], [66]. iDTPnd might be a good platform for drug companies to test the interactions of novel drugs with different CYPs.

**Table 5:** **Interactions between kinase inhibitors and CYPs.**

**Drug**	**Subtype**	**Docking score**	**IC50 (**μM**)**
Dasatinib	CYP2E1	−194.61	>50
Dasatinib	CYP2A6	−100.57	35
Pazopanib	CYP1A2	−105.97	16

*Note*: These interactions are also reported in the FDA Orange Books [65], [66]. CYP, cytochrome 450; IC50, half maximal inhibitory concentration.

### Comparison of iDTPnd with previous methods

Due to the importance of protein–drug interactions, several computational studies have addressed the problem of identifying novel targets of drugs from different angles. However, most of these studies do not benchmark their performance on known targets and use different datasets, thus making it hard to compare among these studies. Other studies reported low precision values (29%, 30%, and 49%, respectively) [17], [19], [21]. Moreover, it is well established in literature that a dataset of confirmed negative relationships (not the negative dataset generated by randomly sampled drugs and potential targets) is pertinent to the improvement of drug target predictions [67], [68]. Cichonska et al. [33] have used machine learning methods to predict the binding affinities of kinase inhibitors to the kinome. Although the authors reported some success, it was not obvious to choose the kernels and regularization parameters for applying their methodology to new drugs. Moreover, it was surprising that 3D features for both drugs and targets did not improve the performance of their methodology. Here, we showed that the weakly conserved 3D features of the drug-binding sites were sufficient to predict the binding affinity of the kinase inhibitors to the proteins whose 3D structures have been resolved. Merget et al. [30] used machine learning to develop a kinase profiling method. Although they reported considerable success (area under the curve > 0.7), the authors did not experimentally validate new predictions. Al-Ali et al. [69] combined cell-based screening with machine learning to correlate the kinase inhibition profile to neurite growth. This investigation had relative specificity for neuronal cells and required more intensive cell-based screening.

Herein, we proposed iDTPnd, a computational method for large-scale discovery of novel targets of known drugs. Our method had the following advantages: 1) it incorporated a negative structural image into the probabilistic scoring function, increasing the sensitivity from 31% to 52% (cut-off = 0.85 as mentioned in [38]); 2) it provided a docking-based interaction score and a measure of the statistical significance of the interaction score, enabling us to identify especially promiscuous small molecules like gefitinib; and 3) the performance of the scoring function was supported by *in vitro* binding experiments that validated 10 predicted interactions. Moreover, we also compared our model with recently published studies of Zhou and colleagues [17] and Luo and colleagues [34]. We analyzed the predicted targets of kinase inhibitors in our dataset by Dr. PRODIS [17] and DTINet [34]. It is important to note that we cannot ensure training/testing data split on these tools and hence the reported results can be considered as a best-case scenario. Dr. PRODIS predicted 7469, 6483, 6263, and 7394 targets for sorafenib, imatinib, dasatinib, and sunitinib, respectively, and did not give any results for pazopanib. Similarly, DTINet predicted 2966 targets for sorafenib, imatinib, dasatinib, and sunitinib, respectively, but did not give any results for pazopanib. We analyzed the top 50 and top 200 targets for each drug predicted by Dr. PRODIS and DTINet for the known proteins that contain a kinase domain and interact with the respective drugs. As shown in Table 6, the sensitivities of Dr. PRODIS and DTIN were much lower than that of iDTPnd.

**Table 6:** **Comparison of iDTPnd with Dr. PRODIS and DTINet**

**Drug**	**Dr. PRODIS**		**DTINet**	
	**Sensitivity for top 50 predicted targets**	**Sensitivity for top 200 predicted targets**	**Sensitivity for top 50 predicted targets**	**Sensitivity for top 200 predicted targets**
Sorafenib	18%	7.5%	14%	3.5%
Dasatinib	10%	21.5%	20%	5%
Imatinib	14%	27.5%	18%	4.5%
Sunitinib	14%	8%	16%	4%
Pazopanib	---	---	---	---
Average	14%	16%	17%	4.3%

### Application to allosteric binding sites

ATP-binding site has conserved features across most kinase domains, and several kinase inhibitors interact with the human kinome broadly and are not very selective. However, type IV inhibitors bind to allosteric sites that are topologically and spatially distinct from conserved ATP-binding sites. It is natural to extend iDTPnd to allosteric biding sites. However, allosteric binding sites are significantly different from non-allosteric binding sites in terms of shape and residue conservation [70]. The construction of the structural signature requires at least 50% conservation for each position in the signature. Therefore, we plan to explore the application of iDTPnd in detail on allosteric binding sites in future studies.

## Conclusion

In this study, we developed a computational model iDTPnd to discover the novel targets of known drugs. For the five kinase inhibitors in our dataset, we identifed the known targets with 52% sensitivity and 55% specificity. The predictive capability of iDTPnd was supported by the validation of top predicted targets using *in vitro* binding experiments. First, we showed that 4 of the top 10 predicted targets of sorafenib were binders. PKCη and MAPKAPK2 had Kd values similar to the primary targets of sorafenib. It is therefore possible that these interactions can be exploited in various cancer treatments. Similarly, the interaction between sorafenib and MHC class I proteins might play currently unexplored roles in immune response to kinase inhibitors. Previously, abacavir, an HIV protease inhibitor, has been shown to alter the peptide binding preference of MHC class I proteins. It is probable that same might be true for several kinase inhibitors. Second, we verified kinase inhibitor interactions with two proteins (ERα and CDK2) that appeared in the top 10 predicted target list of more than one kinase inhibitors. In both cases, our predicted interactions were verified by *in vitro* experiments. Beyond validating our predictions, experimental results also suggest that our method can serve as a platform for kinase inhibitor combination studies. The experimental validation showed that our false positive rate is very low compared with other studies. The false negative rate can be improved in future studies by incorporating structure-independent information like expression data from GTEx and ENCODE projects. Our method is generic and can be used broadly for all types of small molecule drugs for which sufficient 3D structures of known targets (∼30) have been solved.

## Code availability

The code for constructing the structural signature is available at https://sfb.kaust.edu.sa/Documents/iDTP.zip.

## Competing interests

The authors have declared no competing interests.

## CRediT authorship contribution statement

**Hammad Naveed:** Conceptualization, Methodology, Investigation, Software, Writing – review & editing, Writing – review & editing. **Corinna Reglin:** Validation. **Thomas Schubert:** Validation. **Xin Gao:** Supervision, Investigation, Writing – review & editing. **Stefan T. Arold:** Investigation, Writing – review & editing. **Michael L. Maitland:** Investigation, Writing – review & editing.

## Supplementary material

Supplementary Figure S1 **Flow chart of the methodology**

Supplementary Table S1 **Dataset**

Supplementary Table S2 **Performance using positive signature alone**

Supplementary Table S3 **Positive and Negative Signature**

Supplementary Table S4 **Predicted Targets of Dasatinib**

Supplementary Table S5 **Predicted Targets of Imatinib**

Supplementary Table S6 **Predicted Targets of Sunitinib**

Supplementary Table S7 **Predicted Targets of Pazopanib**
