# Supplementary material for: Identifying Novel Drug Targets by iDTPnd: A Case Study of Kinase Inhibitors
Source: Genomics Proteomics Bioinformatics. 2021 Mar 29;19(6):986–97. doi: 10.1016/j.gpb.2020.05.006 (PMC9403029; doi:10.1016/j.gpb.2020.05.006)
Supplement: Supplementary Table S3 — Positive and Negative Signature [file mmc4.docx]

**Table S3 The number of structures used to make the positive and negative signatures and the redundancy cut-off for each signature.**

|  | **Positive signature** | **Preservation ratio (%)** | **Negative signature** | **Preservation ratio (%)** |
| --- | --- | --- | --- | --- |
| Sorafenib | 31 | 50 | 90 | 60 |
| Sunitinib | 93 | 60 | 60 | 60 |
| Dasatinib | 52 | 60 | 111 | 65 |
| Imatinib | 31 | 50 | 117 | 50 |
| Pazopanib | 46 | 60 | 111 | 65 |
